# The role of mAKAPβ in the process of cardiomyocyte hypertrophy induced by angiotensin II

**DOI:** 10.3892/ijmm.2015.2119

**Published:** 2015-03-02

**Authors:** HUIXIN GUO, BAOXIN LIU, LEI HOU, ERLINDA THE, GANG LI, DONGZHI WANG, QIQIANG JIE, WENLIANG CHE, YIDONG WEI

**Affiliations:** Department of Cardiology, Shanghai Tenth People’s Hospital, Tongji University School of Medicine, Shanghai 200072, P.R. China

**Keywords:** angiotensin II, cardiomyocyte hypertrophy, mAKAPβ, A-kinase anchoring protein, extracellular signal-regulated kinase

## Abstract

Angiotensin II (AngII) is the central product of the renin-angiotensin system (RAS) and this octapeptide contributes to the pathophysiology of cardiac hypertrophy and remodeling. mAKAPβ is an A-kinase anchoring protein (AKAP) that has the function of binding to the regulatory subunit of protein kinase A (PKA) and confining the holoenzyme to discrete locations within the cell. In this study, we aimed to investigate the role of mAKAPβ in AngII-induced cardiomyocyte hypertrophy and the possible mechanisms involved. Cultured cardiomyocytes from neonatal rats were treated with AngII. Subsequently, the morphology of the cardiomyocytes was observed and the expression of mAKAPβ and cardiomyocyte hypertrophic markers was measured. mAKAPβ-shRNA was constructed for RNA interference; the expression of mAKAPβ and hypertrophic markers, the cell surface area and the [^3^H]Leucine incorporation rate in the AngII-treated rat cardiomyocytes were detected following RNA interference. Simultaneously, changes in the expression levels of phosphorylated extracellular signal-regulated kinase (p-ERK)2 in the cardiomyocytes were assessed. The cell size of the AngII-treated cardiaomyocytes was significantly larger than that of the untreated cardiomyocytes. The expression of hypertrophic markers and p-ERK2, the cell surface area and the [^3^H]Leucine incorporation rate were all significantly increased in the AngII-treated cells. However, the expression of mAKAPβ remained unaltered in this process. RNA interference simultaneously inhibited the protein expression of mAKAPβ and p-ERK2, and the hypertrophy of the cardiomyocytes induced by AngII was attenuated. These results demonstrate that AngII induces hypertrophy in cardiomyocytes, and mAKAPβ is possibly involved in this process. The effects of mAKAPβ on AngII-induced cardiomyocyte hypertrophy may be associated with p-ERK2 expression.

## Introduction

Cardiac hypertrophy is one of the characteristics of hypertension, myocardial infarction, valvular disease, cardiomyopathy and congestive heart failure. It is a compensatory response of cardiomyocytes to the changes in the molecular and biochemical microenvironment, including pressure overload and chronic increases in circulating hormones that place long term stress on the heart (1,2). When the ventricle is initially stressed, cardiac function can be maintained by adaptive myocyte hypertrophy, since an adequate number of mast cells can meet the requirements of the increasing ventricular wall tension and improve cardiac output. Moreover, neurohumoral factors stimulated by the adaptive hypertrophic growth of the myocardium and the activation of intracellular signaling pathways in the heart and vasculature can also meet the metabolic demands of the body. However, excessive cellular hypertrophy and long-term hemodynamic overload will finally lead to maladaptive myocardial remodeling and the depression of myocardial contractility, which can progress to congestive heart failure, a leading cause of morbidity and mortality in our society (3,4).

Myocardial remodeling and the transition from compensated hypertrophy to the failure of the myocardium involve a complex series of events at the molecular and cellular level, which may result in changes in myocardial structure and function (5). To date, therapy for heart failure is generally palliative. Thus, a better understanding of the intracellular signal transduction networks that control myocyte cell growth may provide new therapeutic directions. Signal transduction networks are composed of second messengers, enzymes and ion channels that form pathways relaying specific signals from the cell surface to intracellular organelles (6). Multiple signaling pathways control the induction of myocyte hypertrophy. These pathways include the cAMP-dependent protein kinase A (PKA) pathways, the mitogen-activated protein kinase (MAPK) pathways and the calcineurin (CaN)-nuclear factor of activated T cells (NFATc) transcription factor pathway (7,8). These pathways serve as an important link between pathological factors in the environment and the changes in the structure and function of the cellular elements of the myocardium. An emerging concept in the field of signal transduction is the existence of nodes within a network where multiple signaling pathways converge and share common molecules, thereby facilitating crosstalk between pathways (6,9,10). Molecules that participate in these centers of integration are of special therapeutic interest due to their potential role in the coordinated modulation of multiple signaling pathways.

A recent attractive family of scaffolding molecules in the myocardium is the A-kinase anchoring protein (AKAP) family (11,12). One member of this family that is involved in cytokine and adrenergic-induced hypertrophy is the alternatively spliced isoform of muscle AKAP (mAKAPβ) expressed exclusively in striated myocytes (13). Known for its ability to bind to PKA, mAKAPβ also binds to multiple proteins, including exchange factor activated by cAMP (Epac), adenylate cyclase 5 (14), cAMP-specific phosphodiesterase 4D3 (PDE4D3), phosphatidylinositol-specific phospholipase Cε (PLCε) (2), extracellular signal-regulated kinase (ERK)5 (13), protein phosphatase 2A (PP2A) (12), the cardiac-specific type II ryanodine receptor (RyR2) (15), the Ca^2+^/calmodulin-dependent protein phosphatase calcineurin Aβ (CaN, PP2B) (16), the sodium/calcium exchanger, NCX1 (17), hypoxia-inducible factor 1α (HIF1α) and ubiquitin E3-ligases involved in HIF1α regulation (18) and myopodin (19). mAKAPβ localizes to the nuclear envelope of myocytes, where it is targeted by nesprin-1α (20).

The renin-angiotensin system (RAS) functions as a neuro-endocrine system playing a key role in cardiovascular and renal physiology. The overactivation of the RAS system has been implicated in the induction and progression of hypertension, atherosclerosis, cardiac hypertrophy, heart failure, ischemic heart disease and renovascular disorders (21–25). In the RAS system, angiotensinogen is converted to angiotensin I (Ang I) under the catalytic activity of renin and is subsequently converted to angiotensin II (AngII), a short-chain octapeptide, in the presence of Ang I-converting enzyme (ACEI). AngII, as the principal component of the RAS cascade, mediates the physiological control of blood pressure and electrolyte balance through a variety of effects that affect the function of most of the organs, including the heart, kidneys, adrenal glands, vasculature and the central nervous system. Studies have demonstrated that AngII plays an important role in cardiac cell proliferation, apoptosis and inotropy (26,27). However, chronic stimulation or overactivation produces deleterious effects on cardiovascular and renal function (22,23).

Although there have been several previous studies detailing the role of AngII or mAKAPβ in the progression of cardiocyte hypertrophy (13,16,26,27), whether mAKAPβ, as a key downstream molecule in multiple signal transduction pathways, is involved in the process of AngII-induced cardiocyte hypertrophy and its potential role remain unclear. The aim of this study was to clarify the role of mAKAPβ in AngII-induced cardiomyocyte hypertrophy and investigate the possible mechanisms involved.

## Materials and methods

### Animals and reagents

All the experiments were performed in accordance with the Guidelines of Animal Experiments from the Committee of Medical Ethics at the National Health Department of China (Shanghai, China) and were approved by the Central Laboratory of the Shanghai Tenth People’s Hospital (Shanghai, China). The suppliers of the materials and reagents were as follows: i) animals: Kunming male rats (Shanghai SLAC Laboratory Animal Co., Ltd., Shanghai, China); ii) equipment: high-speed refrigerated centrifuge (Heraeus, Hanau, Germany), real-time PCR apparatus (Thermocycler; Biometra, Göttingen, Germany), gel image processing system (Media Cybernetics, Inc., Rockville, MD, USA), liquid scintillation counter (Beckman Coulter, Brea CA, USA); iii) reagents: Dulbecco's modified Eagle’s medium (DMEM; Gibco, Grand Island, NY, USA), fetal bovine serum (FBS; GE Healthcare Life Sciences HyClone Laboratories, Logan, UT, USA), AngII (Sigma-Aldrich Co., St. Louis, MO, USA), [^3^H]Leucine (China Institute of Atomic Energy, Beijing, China), RNA PCR kit (Takara Bio Inc., Otsu, Japan), reverse transcriptase (Promega Corp., Madison, WI, USA), anti-GADPH antibody (Cat. no. G8795; Sigma-Aldrich Co.), anti-mAKAPβ antibody (Cat. no. 07-087; Merck Millipore, Billerica, MA, USA), phosphorylated (p-)ERK2 antibody (Cat. no. #9108; Cell Signaling Technology, Beverly, MA, USA), horseradish peroxidase-conjugated secondary antibody (Cat. no. SP-9002; Zhongshan Golden Bridge Biotechnology Co., Ltd., Beijing, China.), pLvx-shRNA2 vector (Invitrogen Corp., Madison, WI, USA), DH-5α *E. coli* competent cells (Life Technologies, Grand Island, NY, USA) and the 293T cell line [American Type Culture Collection (ATCC), Manassas, VA, USA].

### Cell culture and identification

Cardiomyocytes were isolated from 2- to 3-day old male Kunming rats under sterile conditions. In brief, the hearts were excised from the animals, cut into 1 mm^3^ fragments and then rinsed in Hanks' balanced salt solution prior to digestion with 3 rounds of collagenase type II (Life Technologies) in Hanks’ balanced salt solution without Ca^2+^ and Mg^2+^ with ice-cold phosphate-buffered saline (PBS) solution. The extracts were then filtered and centrifuged at 1,000 × g for 5 min. The clear supernatants containing ventricular cells were collected. All the cells were cultured in DMEM (Gibco), which contained 5 mM glucose, and were supplemented with 10% FBS and 1% penicillin/streptomycin at 37°C with 5% CO_2_ in a humidified atmosphere for 24 h. Non-myocytes were removed by the preplating of the cells for 1 h at 37°C. The cardiomyocytes were cultured in DMEM as described above containing 10 *μ*mol/l cytosine arabinoside. Cytosine arabinoside (10 *μ*mol/l) was applied for the first 3 days. Finally, the cultured cells were identified with α-actin immunofluorescence staining, as previously described (28).

### Establishment of cardiomyocyte hypertrophy

The neonatal rat cardiomyocytes were divided into 4 groups as follows: i) control group; ii) 10^−8^ mol/l AngII group; iii) 10^−6^ mol/l AngII group and iv)10^−4^ *μ*mol/l phenylephrine (Phe) group. The cells were prepared for the analysis of the expression of cardiomyocyte hypertrophic markers [atrial natriuretic peptide (ANP) and actin, alpha 1 (Acta1)] 48 h and 72 h after treatment. The optimal concentration and time required to induce cell hypertrophy were identified and used as standard conditions in the following experiments.

### Reverse transcription-quantitative PCR (RT-qPCR) for the detection of the mRNA expression of cardiomyocyte hypertrophic markers

Total RNA was extracted using 1 ml TRIzol reagent (Takara Bio Inc.) according to the manufacturer’s instructions. Total RNA (1 *μ*g) was reverse transcribed using the RT-qPCR kit according to the manufacturer’s instructions. A Mastercycler (Eppendorf AG, Hamburg, Germany) and a SYBR real-time PCR kit (Takara Bio Inc.) were used for qPCR. The expression values were normalized to those of the unaltered control, GAPDH. All the RT-qPCR reactions were carried out in triplicate within each experiment, and the experiments were replicated at least 3 times. The primers used to amplify GADPH, ANP and Acta1 were as follows: GAPDH upstream, 5′-TGCCCAGAACATCATCCCT-3′ and downstream, 5′-GGTCCTCAGTGTAGCCCAAG-3′; ANP upstream, 5′-TGGATACACTGGCATCTACT-3′ and downstream, 5′-TCTTCACCGTCCTCCTCAA-3′; and Acta1 upstream, 5′-AAGTACTCCGTGTGGATCGG-3′ and downstream, 5′-GGGCAGCAGCAACGCA-3′.

### Immunocytochemistry and cell area measurements

The cells were placed in 6-well plates at a density of 1.0×10^5^ cells/ml and 2 ml/well. Following treatment with AngII, the cardio-myocytes were fixed in 4% paraformaldehyde, permeabilized with 0.1% Nonidet P-40, and immunostained with mAKAPβ antibody and α-actinin antibody (Cat. no. sc-17829; Santa Cruz Biotechnology, Inc., Santa Cruz, CA, USA; in order to distinguish the cardiomyocytes from the contaminating fibroblasts) in PBS [0.1% Nonidet P-40 and 3% bovine serum albumin (BSA)] followed by Alexa fluorescent dye-conjugated specific secondary antibody and Hoechst 33258 DNA staining, as previously described (29). Fluorescent images were taken using a Leica DMRA fluorescence microscope (Leica Microsystems GmbH, Wetzlar, Germany) and the cell surface areas were calculated using the NIH ImageJ image processing program. Data were pooled from 2 independent experiments.

### [^3^H]Leucine incorporation

The cardiocytes were placed in 24-well plates at a density of 2×10^5^ cells/ml and 500 *μ*l/well. [^3^H]Leucine (1 *μ*Ci/ml) was used to label the newly translated proteins for 48 h in the absence or presence of AngII. Cells were washed twice with ice-cold PBS and incubated in 5% trichloroacetic acid (TCA) for 30 min at 4°C to precipitate the protein. The precipitates were washed twice and solubilized in 0.4 N NaOH for 2 h. The TCA precipitable radioactivity was measured by scintillation counting. For analysis, the data were pooled from 2–3 independent experiments performed in triplicate.

### mAKAPβ-shRNA lentiviral vector construction and preparation

Primers for mAKAPβ were designed using Oligo 6.0 software (Molecular Biology Insights, Inc. Cascade, CO, USA), which was conducted by Shanghai Sangon Biological Engineering Technology & Services Co., Ltd. (Shanghai, China). The sequences of the primers were as follows: mAKAPβ upstream, 5′-GATCCGACGAACCTTCCTTCCGAATTCAAGAGATTCGGAAGGAAGGTTCGTCTTTTTTG-3′ and downstream, 5′-AATTCAAAAAAGACGAACCTTCCTTCCGAATCTCTTGAATTCGGAAGGAAGGTTCGTCG-3′. A target sequence (5′-GACGAACCTTCC TTCCGAA-3′) was selected to construct the lentiviral shRNA according to a previous study (10). The target sequence was used to design 2 complementary oligonucleotides. Following the dilution of the oligonucleotide fragments, double-stranded DNA fragments were formed in the annealing reaction system. The pLvx-shRNA2 vector was linearized by B*am*HI and E*co*RI restriction enzyme digestion. Pure linearized vector fragments, double-stranded DNA fragments and vector fragments were collected and combined together during a 12-h reaction. The recombinant vector loop was then transformed into the freshly prepared *E. coli* competent cells. Following *E. coli* cell culture for 16 h at 37°C, bacterial colonies were randomly selected as PCR templates. Verification of the positive clones was conducted using PCR technology. The positive purified lentiviral shRNA-expressing plasmids were transfected with the packaging plasmids into 293T cells. After 8 h, the culture medium was changed to complete medium. The supernatant was concentrated and collected following culture for 48 h. The viral titer of the 293T cells was generally up to 10^8^ TU/ml. The transfected cells were stored in a −80°C refrig erator for later use. The exponentially growing cardiomyocytes were seeded at 2×10^5^ cells/well in 1 ml of culture medium into a 12-well plate, and 10 *μ*l of concentrated vector stock with a multiplicity of infection (MOI) value of 20 plus 5 *μ*g/ml polybrene were also added. The cells were then stored in a 37°C incubator. Following overnight incubation, the culture medium was changed to fresh culture medium. The transfection rate was determined by western blot analysis.

### Protein extraction and western blot analysis

Total proteins were obtained by rinsing the treated cells with ice-cold PBS, and lysing in lysis buffer (10 mM Tris pH 7.4, 20 mM NaCl, 5 mM MgCl_2_, 0.5% Nonidet P-40 and 0.1 mM PMSF). The extracts were then centrifuged at 12,000 × g for 10 min at 4°C, and the clear super-natants containing total protein were collected. The protein concentrations were measured using the BCA protein assay kit (Beyotime Institute of Biotechnology, Shanghai, China). Equal amounts of protein were loaded, separated by SDS-PAGE and transferred onto nitrocellulose membranes. After blocking with 5% non-fat milk in Tris-buffered saline with Tween-20 (TBST) at room temperature, the membranes were incubated overnight at 4°C with the previously mentioned primary antibodies. After being incubated with the respective secondary antibodies, immune complexes were detected using the Odyssey system (LI-COR Biosciences, Lincoln, NE, USA) based on the protocol. The band density value was quantified using the NIH ImageJ image processing program.

### Statistical analysis

Data analysis was conducted using the SPSS 16.0 software package for Windows (SPSS Inc., Chicago, IL, USA). Continuous variables are presented as the means ± standard deviation (SD). The statistical significance of the differences was determined by the analysis of variance (ANOVA) or an unpaired two-tailed t-test; a value of P<0.05 was considered to indicate a statistically significant difference.

## Results

### Morphology and identification of cardiomyocytes

**Figure 1:**
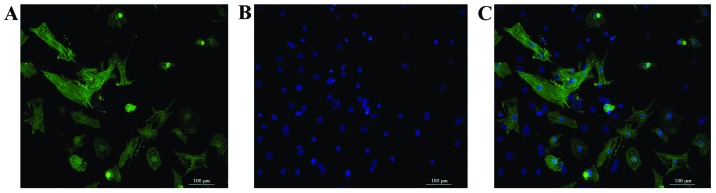
The morphology and identification of cardiomyocytes. (A) Green fluorescence represents cardiomyocytes with α-actinin inmmunofluorescence staining. (B) Blue fluorescence represents cell nucleus detected by Hoechst 33258 staining. (C) Merged inmmunofluorescence staining. The normal cultured cardiomyocytes were positive for α-actinin inmmunofluorescent staining. The negatively stained α-actinin inmmunofluorescent cells indicated the contaminating fibroblasts.

The morphological characteristics of the cardiomyocyte were observed and images were acquired by digital fluorescence microscopy (Fig. 1). The normal cultured cardiomyocytes were positive for α-actinin inmmunofluorescent staining, and were attached, with an irregular or triangular shape. The spontaneous pulsation of the cardiomyocytes was also detected. The negatively stained α-actinin inmmunofluorescent cells were considered as the contaminating fibroblasts.

### Increase in the expression of cardiomyocyte hypertrophic makers at optimal concentrations of AngII and at specific time points following treatment

**Figure 2:**
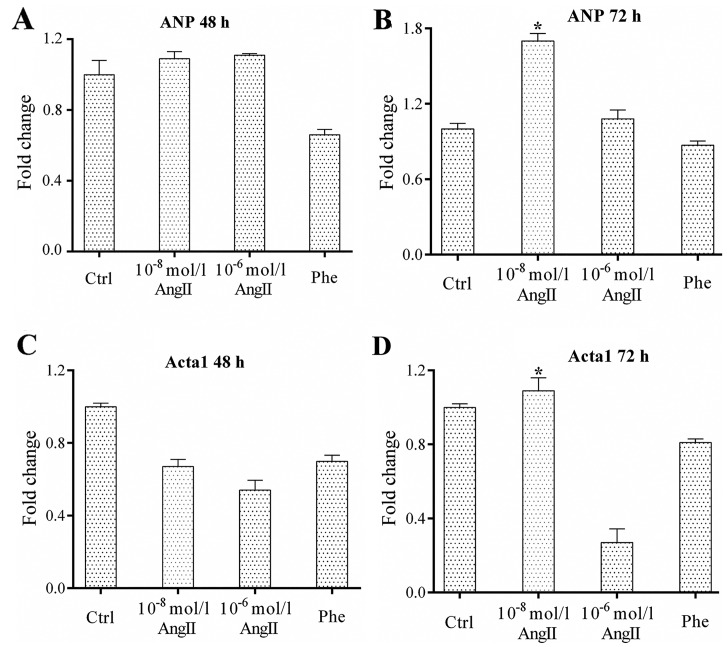
Expression of hypertrophic markers in cardiomyocytes treated with angiotensin II (AngII) at different concentrations and different periods of time. (A and B) Results of assays of atrial natriuretic peptide (ANP) expression in cardiomyocytes incubated with AngII at the designated concentrations (10^−8^ and 10^−6^ mol/l) and different periodes of time (48 and 72 h) by RT-qPCR. (C and D) Results of assays of Acta1 expression in cardiomyocytes incubated with AngII at the designated concentrations (10^−8^ and 10^−6^ mol/l) and different periodes of time (48 and 72 h) by RT-qPCR. The expression of cardiomyocyte hypertrophic markers was increased most when incubated with 10^−8^ mol/l AngII for 72 h. Experiments were repeated at least 3 times. Data are expressed as the means ± SD in the corresponding bar graph and statistical significance was determined by the Student’s t-test. Ctrl, control; Phe, phenylephrine. Columns, mean; error bars, ± SD; ^*^P<0.05.

The effects of AngII (10^−8^ and 10^−6^ mol/l AngII) on the expression of cardiomyocyte hyper-trophic makers at 2 different time points (48 and 72 h) were examined. Our results revealed that AngII at the appropriate concentration increased the levels of the cardiomyocyte hypertrophic makers, ANP and Acta1, at specific time points. The results from RT-qPCR (shown in Fig. 2) revealed that the expression of the cardiomyocyte hypertrophic makers was significantly higher following treatment with 10^−8^ mol/l AngII at 72 h (all P<0.05 vs. control). Thus, treatment of the cells with 10^−8^ mol/l AngII for 72 h may be considered optimal for the induction of cardiomyocyte hypertrophy.

### Role of mAKAPβ expression in the process of AngII-induced cardiomyocyte hypertrophy

**Figure 3:**
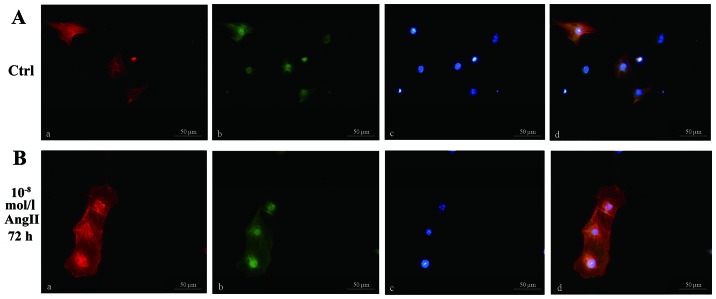
Representative results of morphological analysis of cardiomyocytes treated with or without 10^−8^ mol/l angiotensin II (AngII) for 72 h. (A) Morphology of the cardiomyocytes in the control group (without incubation with AngII). (B) Morphology of the cardiomyocytes treated with 10^−8^ mol/l AngII for 72 h. (a) α-actinin inmmunofluorescence staining, (b) mAKAPβ inmmunofluorescence staining, (c) Hoechst 33258 staining, (d) merged inmmunofluorescence staining. The cardiomyocytes treated with 10^−8^ mol/l AngII for 72 h appeared to be more hypertrophic. mAKAPβ was localized to the nuclear envelope of the cardiomyocytes. Ctrl, control.

**Figure 4:**
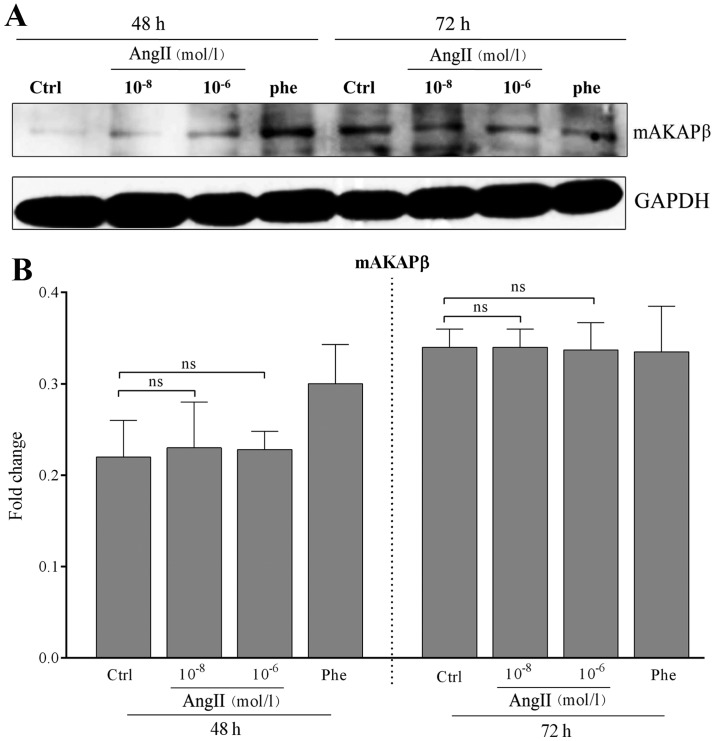
Expression of mAKAPβ in cardiomyocytes treated with angiotensin II (AngII) at different concentrations and for different periods of time. (A) Representative results of assays of mAKAPβ and GAPDH abundance in cardiomyocytes treated with AngII at different concentrations and different periods of time by western blot analysis. (B) The protein expression levels of mAKAPβ and GAPDH were analyzed by western blot analysis using polyclonal antibodies to mAKAPβ and GAPDH to quantify the expression in these groups. No statistically significant differences were observed in the expression of mAKAPβ between the controls and the cells treated with AngII at the designated concentration (all P>0.05). GAPDH was used as an equal loading control. The band value was quantified by densitometric analysis. Experiments were repeated at least 3 times. Data are expressed as the means ± SD in the corresponding bar graph and statistical significance was determined by the Student’s t-test. Ctrl, control; Phe, phenylephrine; ns, not significant. Columns, mean; error bars, ± SD.

The size of the cardiomyocytes treated with or without AngII is shown in Fig. 3. The results revealed that AngII promoted the process of cardiomyocyte hypertrophy. mAKAPβ was localized to the nuclear envelope of the myocytes; however, the expression of mAKAPβ in the AngII-treated groups was similar to that of the control group according to the results of western blot analysis (all P>0.05 vs. control; Fig. 4). These results demonstrated that mAKAPβ was involved in the process of AngII-induced cardiomyocyte hypertrophy; however, its expression remained unaltered.

### RNA interference significantly reduces the expression of mAKAPβ in AngII-induced cardiomyocyte hypertrophy

**Figure 5:**
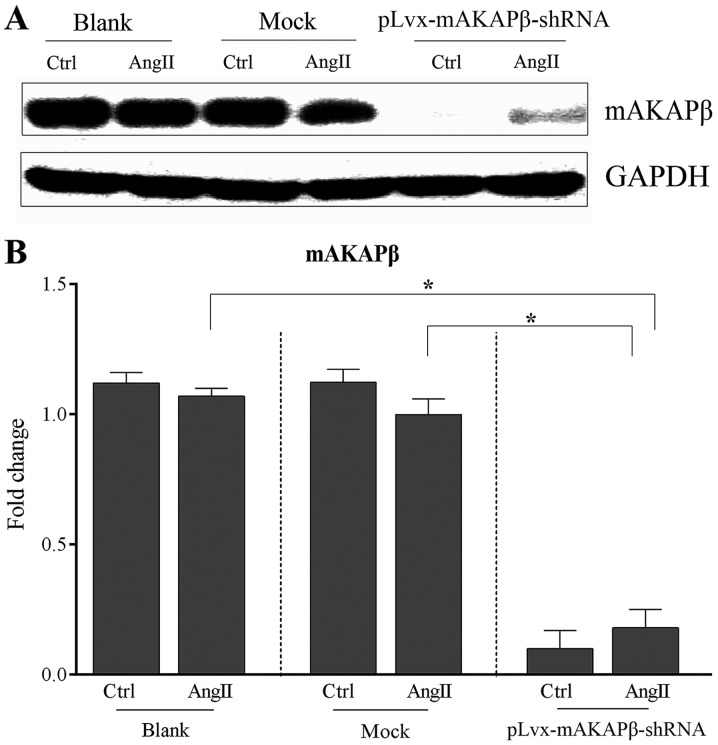
Expression of mAKAPβ in cardiomyocytes following the different treatments. Six sets of cardiomyocytes were divided into 3 groups: i) blank group, no virus; ii) mock group, control virus, and iii) pLvx-mAKAPβ-shRNA lentivirus group. (A) Representative results of assays of mAKAPβ and GAPDH abundance in the 3 groups by western blot analysis. (B) The protein expression levels of mAKAPβ and GAPDH were analyzed by western blot analysis using polyclonal antibodies to mAKAPβ and GAPDH to quantify the expression in these groups. Following treatment with angiotensin II (AngII), the expression of mAKAPβ in the pLvx-mAKAPβ-shRNA lentivirus group was significantly lower when compared with that of the AngII-treated cells in the blank and mock groups (both P<0.05). No statistically significant differences was observed in the expression of mAKAPβ in the blank and mock groups following treatment with AngII compared with the controls (P=0.08 and P=0.06, respectively). GAPDH was used as an equal loading control. The band value was quantified by densitometric analysis. Experiments were repeated at least 3 times. Data are expressed as the means ± SD in the corresponding bar graph and statistical significance was determined by the Student’s t-test. Ctrl, control. Columns, mean; error bars, ± SD; ^*^P<0.05.

The requirement for mAKAPβ in AngII-induced cardiomyocyte hypertrophy was examined by RNA interference using mAKAPβ-shRNA which was constructed as aforementioned (see Materials and methods). Six sets of cardiomyocytes were divided into 3 groups: i) the blank group, no virus; ii) the mock group, control virus; and iii) the pLvx-mAKAPβ-shRNA lentivirus group. After 24 h, the culture medium was changed to maintenance medium which does not contain serum. One set from each group was treated with 10^−8^ mol/l AngII, and the other set was used as a control. All myocardial cells were then cultured for 72 h. The results from western blot analysis revealed that the expression of mAKAPβ in the blank and mock groups showed no statistically significant difference following treatment with AngII compared with the control (P=0.08 and P=0.06, respectively; Fig. 5). However, the expression of mAKAPβ was significantly reduced in the pLvx-mAKAPβ-shRNA lentivirus group following RNA interference. Moreover, even following treatment with AngII, the expression of mAKAPβ in the pLvx-mAKAPβ-shRNA lentivirus group was significantly lower when compared with that of the AngII-treated cells in the blank and mock groups (both P<0.05; Fig. 5).

### mAKAPβ possibly contributes to cardiomyocyte hypertrophy following treatment with AngII

**Figure 6:**
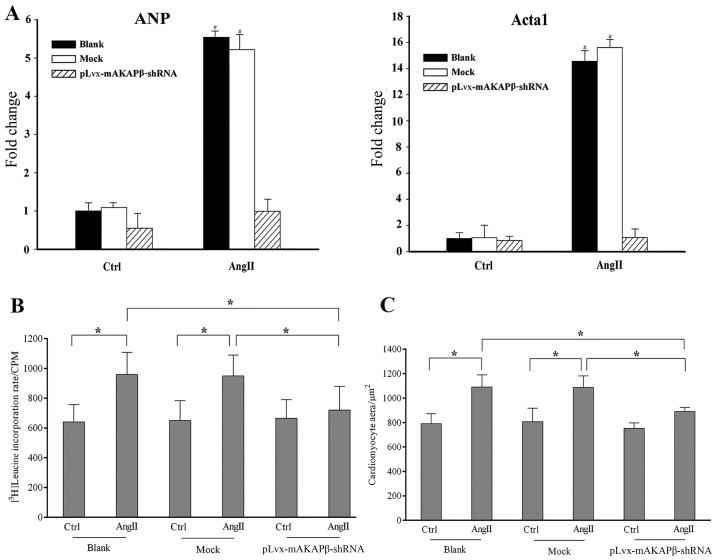
Results of the expression of cardiomyocyte hypertrophic makers, [^3^H]Leucine incorporation rate and cell area measurements of different treatments. Six sets of cardiomyocytes were divided into 3 groups: i) Blank group: no virus, ii) Mock group: control virus, and iii) pLvx-mAKAPβ-shRNA lentivirus group. (A) Results of assays of cardiomyocyte hypertrophic markers expression in different groups. (B) [^3^H]Leucine incorporation rate of each group. (C) Cell surface areas were calculated using the NIH ImageJ image processing program. The expression of cardiomyocyte hypertrophic makers (ANP and Acta1) in blank and mock groups was significantly elevated following the treatment of 10^−8^ mol/l Angiotensin II (AngII) for 72 h (both P<0.05 vs. control). No significant difference was detected in the expression of ANP and Acta1 in the pLvx-mAKAPβ-shRNA lentivirus group compared with the control (P=0.06, P=0.12, respectively). The [^3^H]Leucine incorporation rate and cell surface areas were significantly increased in blank and mock groups, respectively (all P<0.05 vs. control). No significant difference was observed in [^3^H]Leucine incorporation rate and cell surface areas of pLvx-mAKAPβ-shRNA lentivirus group when compared with control, respectively (P=0.12 and P=0.08, respectively). Experiments were repeated at least 3 times. Data are expressed as the mean ± SD in the corresponding bar graph and statistical significance was determined by the Student’s t-test. Ctrl, Control; AngII, Angiotensin II; Columns, mean; error bars, ±SD; ^*^P<0.05.

To define the potential role of mAKAPβ in AngII-induced cardiomyocyte hypertrophy, we examined the expression of cardiomyocyte hypertrophic makers, the [^3^H]Leucine incorporation rate and the cell surface area in each group. The expression of the cardiomyocyte hypertrophic makers (ANP and Acta1) in the blank and mock groups was significantly elevated following treatment with 10^−8^ mol/l AngII for 72 h (both P<0.05 vs. control; Fig. 6A). However, the expression of ANP and Acta1 in the pLvx-mAKAPβ-shRNA lentivirus group showed no significant difference compared with the control (P=0.06 and P=0.12, respectively; Fig. 6A) subsequent to the inhibition of mAKAPβ.

AngII induced a 50.1 and 46.2% increase in the [^3^H]Leucine incorporation rate in the blank group [967.8±147.9 counts per min (CPM) vs. control: 640.1±118.2 CPM] and mock group (954.1±141.2 CPM vs. control: 652.4±133.0 CPM) groups, respectively (Fig. 6B). Similarly, AngII promoted a 38.0% increase in the cell surface area in the blank group compared with the control (790.2±82.9 *μ*m^2^ vs. 1090.3±99.6 *μ*m^2^), and a 34.8% increase was also detected in the mock group compared with the control (807.4±109.8 *μ*m2 vs. 1088.5±94.4 *μ*m^2^) (Fig. 6C). However, the [^3^H]Leucine incorporation rate in the pLvx-mAKAPβ-shRNA lentivirus group showed only a 8.2% increase following treatment with AngII (665.5±125.4 CPM vs. control: 720.5±160.1 CPM) (Fig. 6B). Similarly, the cells were only 18.4% larger in the pLvx-mAKAPβ-shRNA lentivirus group compared with the control (752.6±45.6 *μ*m^2^ vs. 891.1±33.2 *μ*m^2^) (Fig. 6C). These results indicated that the downregulation of mAKAPβ inhibited AngII-induced cardiomyocyte hypertrophy.

### Inhibition of mAKAPβ suppresses the expression of p-ERK2 in AngII-induced cardiomyocyte hypertrophy

**Figure 7:**
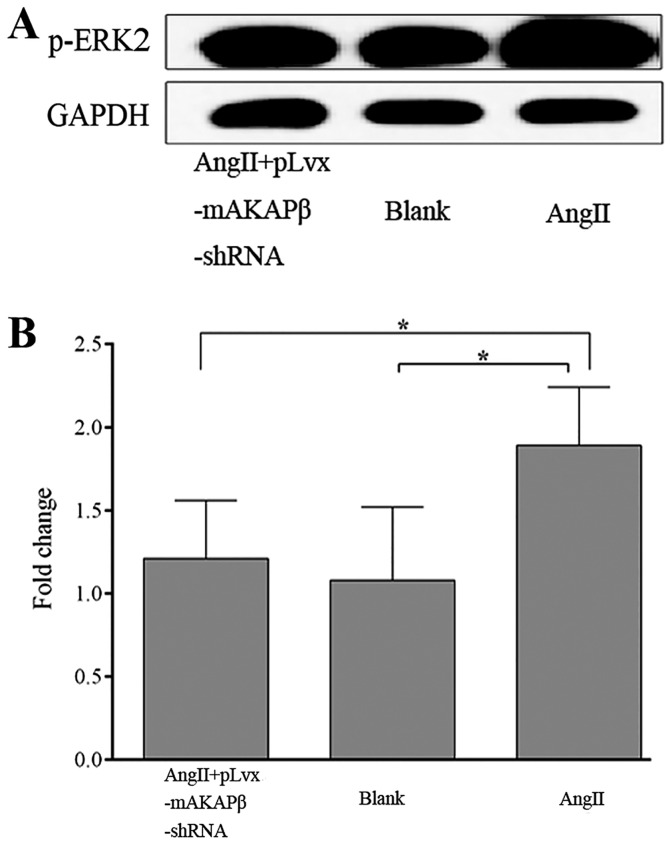
Expression of p-ERK2 in the cardiomyocytes of different treatments. The cells were divided into 3 groups: i) Angiotensin II (AngI I)+pLvx-mAKAPβ-shRNA group; ii) Blank group; iii) AngII group. (A) Representative results of assays of p-ERK2 and GADPH abundances of 3 groups using western blot analysis. (B) The levels of p-ERK2 and GADPH protein expression were analyzed by western blot analysis by using polyclonal antibodies to p-ERK2 and GADPH to quantify the expression in these groups The expression of p-ERK2 was significantly elevated in AngII group, compared with blank group (P=0.009). The expression of p-ERK2 was significantly reduced following the inhibition of mAKAPβ by RNA interference in AngII+pLvx-mAKAPβ-shRNA group, compared with AngII group (P=0.013). No significant difference was observed between the blank group and AngII+pLvx-mAKAPβ-shRNA group (P=0.10).

We also preliminarily investigated the possible mechanisms underlying the effects of the downregulation of mAKAPβ on the process of AngII-induced cardiomyocyte hypertrophy. Since AngII activates ERK in the process of cardiomyocyte hypertrophy (30), we also examined the expression of p-ERK2 in the process of AngII-induced cardiomyocyte hypertrophy. The cells were divided into 3 groups as follows: i) the AngII + pLvx-mAKAPβ-shRNA group; ii) the blank group; and iii) the AngII group. The results from western blot analysis revealed that AngII significantly increased the expression of p-ERK2 (P=0.009 vs. blank group) (Fig. 7). However, the expression of p-ERK2 was significantly reduced following the inhibition of mAKAPβ by RNA interference in the AngII + pLvx-mAKAPβ-shRNA group (P=0.013 vs. AngII group) (Fig. 7). These results indicated that AngII induced an increase in p-ERK2 expression in the process of cardio-myocyte hypertrophy, and this increase may be mediated by mAKAPβ.

## Discussion

The main aim of this study was to investigate the effects of mAKAPβ on AngII-induced cardiomyocyte hypertrophy *in vitro*. The potential mechanisms involved were also explored. RNA interference technology was used and the expression of mAKAPβ in AngII-induced cardiomyocyte hypertrophy was determined. Simultaneously, the effects on the activation of the downstream signaling molecule, ERK2, were also examined. The findings from our study indicated that in the process of cardiomyocyte hypertrophy induced by AngII, the expression of mAKAPβ showed no significant change; however, a sharp increase in p-ERK2 expression was detected, and this increase may be mediated by mAKAPβ.

Previous studies (31–33) have demonstrated that the mechanisms responsible for cardiomyocyte hypertrophy involve the concerted activation of a network of signal transduction pathways. The hypertrophic signaling network is controlled by a wide array of neuroendocrine, paracrine and autocrine hormones which include, for example, catecholamines and peptide hormones that activate G-protein coupled receptors, interleukin (IL)-6 type cytokines that activate gp130 receptors, and growth factors that activate receptor tyrosine kinases. The hypertrophic signaling network promotes protein synthesis and sarcomeric assembly in the cytoplasm and induces stereotypical changes in gene expression in the nucleus, resulting overall in increased myocyte volume and power. The signaling pathways that control these processes are diverse and include a large set of second messengers, protein kinases, phosphatases and downstream effector molecules. There have been a number of elegant studies concerning hypertrophic intracellular signaling (9,34,35). However, it is difficult to provide a comprehensive understanding of this complex process. Thus, many researchers have focused on a recently emerging concept in signal transduction, the idea that scaffold proteins mediate the crosstalk and integration of different signaling pathways through the formation of multimolecular protein complexes that incorporate the components of different pathways (10,19,36).

One particular signaling complex that has been well characterized and implicated in the control of cardiac hypertrophy is the ‘mAKAPβ signalosome’, a concept that has become established over the past decade, since the ‘mAKAPβ signalosome’ is a large multimolecular complex that is involved in multiple signaling transduction pathways, which integrate multiple upstream signals and transduce specific downstream signals through the regulation of multiple effectors (10). mAKAP was initially identified in a screening for PKA binding proteins. mAKAPα and mAKAPβ are the 2 known isoforms encoded by the single mAKAP (AKAP6) gene and are expressed in neurons and striated myocytes (36). As a consequence of alternative mRNA splicing, mAKAPβ is identical to residues 245-2314 (the C terminus) of mAKAPα. In adult and neonatal cardiac myocytes, mAKAPβ is primarily localized to the outer nuclear membrane through its association with nesprin-1α (20). In addition to PKA, many other mAKAPβ anchoring proteins, i.e., ‘mAKAPβ signalosome’ components, have been identified (2,12–19). Due to the association of these proteins with mAKAPβ in cardiaomyocytes, researchers have confirmed that the ‘mAKAPβ signalosome’ plays an important role in the regulation of pathologic cardiomyocyte hypertrophy in response to upstream signals. To date, 3 of these proteins that can induce cardiomyocyte hypertrophy have been identified: ERK5, calcineurin Aβ and PLCε (2,13,16).

Much evidence has indicated that cardiac RAS is linked to the formation of cardiac hypertrophy. Studies have demonstrated that all components of RAS (e.g., renin, angiotensinogen, ACE and AngII receptors) are identified in the heart at both the mRNA and the protein levels (37) and that RAS is activated in experimental left ventricular hypertrophy induced by hemodynamic overload (37–40). It is already known that AngII plays an important role in the regulation of the structure and function in the heart (41,42). Apart from its physiological role, AngII, acting through a family of receptors, exerts an array of diverse pathological effects in the heart, blood vessels, kidneys, adrenal glands, liver, smooth muscle, skeletal muscle, pancreatic islets and other cell types (43–45). AngII directly induces cardiomyocyte hypertrophy even without an increase in vascular resistance or cardiac afterload (38). In fact, *in vitro* studies have demonstrated that pressure overload also causes an increase in AngII levels in cardiaomyocytes of neonatal rats, and AngII may act to promote the growth of cardiomyocytes through an autocrine mechanism (46,47). Moreover, AngII evokes a variety of signals to induce cardiomyocyte hypertrophy and the proliferation of cardiac fibroblasts (46,48), and the AngII-evoked signal transduction pathways differ among cell types (49). It has been demonstrated that AngII increases protein synthesis and induces the pathological hypertrophic growth of cardiomyocytes in an ERK-dependent manner (30). However, few studies have explored the underlying mechanisms involved in this process. The results of the present study demonstrated that the expression of mAKAPβ remained unaltered in the process of cardiomyocyte hypertrophy induced by AngII. However, the expression of p-ERK2 in this process was significantly increased. Furthermore, the expression of p-ERK2 decreased significantly and AngII-induced cardiomyocyte hypertrophy was markedly attenuated following the inhibition of mAKAPβ. Our results mainly provide preliminary evidence that mAPAKβ potentially affects AngII-induced cardiomyocyte hypertrophy through its regulation of p-ERK2, which is useful in the understanding of the molecular mechanisms and the pathogenesis of cardiac remodeling. Our findings may have implications regarding novel therapeutic targets in cardiac hypertrophy.

Although we analyzed the preliminary role of mAKAPβ in the present study, the effects of mAKAPβ in cardiac hypertrophy are believed to be more profound than previously reported. This study was performed using *in vitro* experimental systems. Additionally, the association between mAKAPβ and AngII-induced cardiomyocyte hypertrophy should be confirmed in the isolated heart through *in vivo* experiments. We only detected the role of mAKAPβ through alterations in the ERK pathway. Since the mAKAPβ complex is involved in multiple signaling transduction pathways, further studies are required to elucidate the other signaling pathways involved in this process.

In conclusion, the results of the present study demonstrated that the expression of mAKAPβ remained unaltered in the process of AngII-induced cardiomyocyte hypertrophy, and that the effects of mAKAPβ on cardiomyocyte hypertrophy induced by AngII may be associated with p-ERK2, whose expression was significantly increased in this process.
